# Dexmedetomidine prolongs the duration of local anesthetics when used as an adjuvant through both perineural and systemic mechanisms: a prospective randomized double-blinded trial

**DOI:** 10.1186/s12871-022-01716-3

**Published:** 2022-06-07

**Authors:** Nana Bao, Kejian Shi, YiQuan Wu, Yuting He, Zhengjie Chen, Yuan Gao, Yun Xia, Thomas J. Papadimos, Quanguang Wang, Riyong Zhou

**Affiliations:** 1 Department of Anesthesiology, the First Affiliated Hospital of Wenzhou Medical University, South Baixiang, Wenzhou City, 325000 Zhejiang Province China; 2 Department of Anesthesiology, Zhejiang University School of Medicine Second Affiliated Hospital, Hangzhou, Zhejiang China; 3 Department of Anesthesiology, Zhejiang University School of Medicine Sir Run Run Shaw Hospital, Hangzhou, Zhejiang China; 4 Department of Anesthesiology, Ohio State University Wexner Medical Center, Columbus, OH USA

**Keywords:** Dexmedetomidine, Ropivacaine, Perineural mechanism, Systemic mechanism

## Abstract

**Background:**

To study the respective peripheral and systemic mechanisms of action of dexmedetomidine, as adjuvant to regional anesthesia, we compared dexmedetomidine added to ropivacaine for mid-forearm nerve blocks, to either systemic-only dexmedetomidine, and to a control with no dexmedetomidine.

**Methods:**

Sixty patients undergoing hand surgery were randomly divided into three groups (*n* = 20 per group). Each group underwent a triple-nerve (median, radial and ulnar) mid-forearm blocks with 0.75% ropivacaine. In the DexP group, 60 µg of dexmedetomidine were added to the anesthetic mixture, while in the DexIV group, they were intravenously infused. Normal saline as a placebo was used, either as adjuvant, or intravenously. All patients underwent also a supraclavicular block with 1.5% lidocaine for tourniquet pain. The main outcomes were the duration of analgesia and the duration of sensory blockade separately for each nerve termination of the upper limb, and the duration of motor blockade of the upper limb. Tolerance was assessed by blood pressure and heart rate, and the report of adverse events.

**Results:**

Duration of analgesia was longer in the DexP group, in comparison to the two other groups (*P* < 0.001), while it was similar in the DexIV and the control group. For cutaneous territories targeted by the three mid-forearm blocks, the between-group differences behaved similarly. For the other cutaneous territories (musculocutaneous and posterior brachial cutaneous nerves), duration of sensory blockade was shorter in the control group than in the two dexmedetomidine groups. For duration of motor blockade, the between-group differences behaved similarly. Both blood pressure and heart rate were reduced in the DexP and the DexIV groups, compared to the control.

**Conclusions:**

Dexmedetomidine used as an adjuvant to regional anesthesia may act mostly though a perineural mechanism, especially for the sensory aspects of anesthesia. A systemic action might however explain other clinical effects.

**Trial registration:**

ChiCTR-IOR-17011149, date of registration: 16/04/2017

## Background

Dexmedetomidine is a selective α2 adrenoceptor agonist. Dexmedetomidine has been reported to prolong the duration of the effect of peripheral nerve blocks by both peripheral and intravenous (IV) routes [1–4]. However, the specific mechanism remains unclear. A recent systematic review [1] concluded that moderate quality evidence suggests that IV dexmedetomidine is an inferior peripheral nerve block adjunct compared with perineural dexmedetomidine, thereby indicating a primarily peripheral mechanism of action. This mechanism may also explain the observation of another systematic review [5] that indicated IV dexmedetomidine does not improve the block characteristics. The IV route for administering dexmedetomidine has been suggested [6] to be comparable to the perineural route with respect to onset and duration of blocks as well as the duration of analgesia, but the evidence has been insufficient.

Our previous study [7] showed that an ultrasound-guided mid-forearm nerve block with 0.75% ropivacaine combined with a supraclavicular brachial plexus block using 1.5% lidocaine can provide long-term postoperative analgesia for wrist and hand surgery and that it facilitates the return of motor function in the upper limb. The supraclavicular brachial plexus was blocked with lidocaine to avoid tourniquet pain and benefitted early movement of the upper limb. Mid-forearm nerves were blocked with ropivacaine to ensure prolonged postoperative analgesia. However, the effect of dexmedetomidine on mid-forearm nerve blocks is yet to be elucidated.

Hence, a randomized, double-blinded trial was conducted in which an ultrasound-guided 1.5% lidocaine supraclavicular brachial plexus block was combined with 0.75% ropivacaine mid-forearm nerve blocks (our previous block model) for patients undergoing hand surgery. Dexmedetomidine was either added to ropivacaine or administered IV to unravel the mechanism of action of dexmedetomidine when used as an adjuvant to local anesthetics.

## Methods

### Patients

The Ethics Committee of the First Affiliated Hospital of Wenzhou Medical University approved this prospective trial, and it was registered at the Chinese Clinical Trial Registry (ChiCTR-IOR-17011149, date of registration: 16/04/2017). The study adhered to the World Medical Association Declaration of Helsinki. The data have been furnished in accordance with the Consolidated Standards of Reporting Trials (CONSORT) statement. This article has been presented in accordance with the CONSORT reporting checklist. Sixty patients aged ≥ 18 years belonging to the American Society of Anesthesiologists (ASA) I – III, and who were scheduled for elective hand surgery between June 2018 and October 2018 were enrolled in the study after obtaining written informed consent. Exclusion criteria were as follows: local anesthetic allergies, chronic pain, coagulopathy, infection at the planned injection site, peripheral neurologic disease, and an inability to comprehend the study-related procedures.

### Study design

On arrival at the preoperative area all patients received an IV catheter secured in the opposite forearm (to the site of surgery). Standard monitoring methods included noninvasive blood pressure, electrocardiogram, and pulse oximetry. All patients received 1 mg of IV midazolam and 20 µg of fentanyl before the nerve blockade for sedation and analgesia. An anesthesiologist experienced in ultrasound-guided regional anesthesia who was blinded to the groupings performed all blocks in the preoperative preparation room using an ultrasound machine (SonoSite X-Porte, SonoSite, Bothell, WA, USA) with a 6 – 15 MHz high-frequency linear array transducer. The drug preparation for this study was made by a nurse anesthetist who did not participate in the trial. The drugs included dexmedetomidine (2 mL: 200 µg, 16,101,432, Jiangsu Hengrui Pharmaceutical Co., Ltd., China), lidocaine (5 mL: 0.1 g, H12021000, Tianjin Jinyao Pharmaceutical Co., Ltd., China), and ropivacaine (75 mg/10 mL, LAZS, AstraZeneca, Sweden).

### Conduction of anesthesia

The anesthesia process was as follows: First, for the initiation of the block, patients were administered an infusion with the study IV treatment over 15 min. Subsequently, the supraclavicular brachial plexus block was performed using the study anesthetic mixture. The block was performed under ultrasound-guidance using a double-injection method [8]. Accordingly, 10 mL of the study solution was administered in the “corner pocket” (the junction of the first rib and subclavian artery), and another 10 mL was injected into the neural cluster formed by the trunks and divisions of the brachial plexus. Thereafter, a triple-nerve (median, radial, and ulnar) mid-forearm block was performed. The patients were positioned supine with the operated arm abducted and externally rotated and the palm facing up. The ultrasound probe was placed perpendicular to the middle of the forearm to obtain a short-axis cross-sectional ultrasound image. The median nerve was identified as a round or oval hyperechoic structure located between the flexor digitorum profundus and flexor digitorum superficialis muscles. An ultrasound scan of the ulnar side revealed a round or oval hyperechoic structure medial to the artery, which was identified as the ulnar nerve. A scan of the radial side identified the superficial branch of the radial nerve as a round or oval hyperechoic structure between the radial artery and the radius. After identifying the target nerves with a sterile ultrasound probe, the skin was disinfected, and 3 mL of the study solution was injected around each nerve to ensure circumferential spread.

### Study groups

**Fig. 1:**
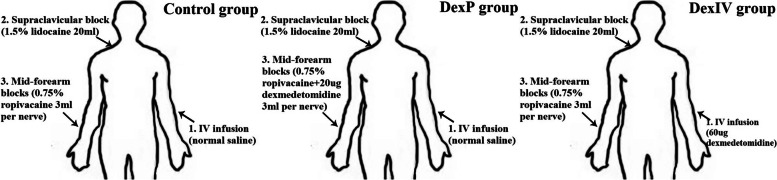
The grouping and flow chart of the experiment

Sixty patients were randomly divided into three groups, namely, DexP group (*n* = 20), DexIV group (*n* = 20), and Control group (*n* = 20), according to the allocation sequences generated by a random number table and delivered in sealed opaque envelopes. Figure 1 depicts the groupings and the flow chart of the experiment. The treatments administered for each group were as follow. The study IV treatment of patients: 100 mL of normal saline was administered to the DexP and Control groups and 100 mL of normal saline mixed with 60 µg of dexmedetomidine was administered to the DexIV group. The study anesthetic mixture for supraclavicular brachial plexus blocks: 20 mL of 1.5% lidocaine for all groups. The study anesthetic mixture for mid-forearm nerve blocks: 0.75% ropivacaine (3 mL of 0.75% ropivacaine + 0.2 mL of saline applied to each nerve) for the DexIV and Control groups and 0.75% ropivacaine mixed with 20 µg of dexmedetomidine (3 mL of 0.75% ropivacaine + 0.2 mL of dexmedetomidine applied to each nerve, 60 µg of dexmedetomidine in total) for the DexP group.

### Assessments and endpoints

After completion of the nerve blocks, the patient was tested for surgical anesthesia. The success of surgical anesthesia was defined as the lack of requirement for additional anesthetic. The need to convert to general anesthesia or any requirement for additional anesthetic was defined as an anesthetic failure. The patients were given 200 mg of celecoxib orally every 12 h for postoperative pain if the Numerical Rating Pain score (NRPS) was > 4.

After surgery, another anesthesiologist who was unaware of the groupings assessed the pain sensation in each nerve distribution based on the pinprick test, motor function of the upper limb, and any adverse events. Sensory block was evaluated using the pinprick test every 30 min after the surgery at the center of the thenar eminence (median nerve), little finger (ulnar nerve), dorsum of the hand over the metacarpophalangeal joint (radial nerve), posterior of the upper arm (posterior brachial cutaneous nerve), and the lateral aspect of the forearm (musculocutaneous nerve). The duration of analgesia was defined as the time from the end of the local anesthetic injection to the recovery of pinprick sensation in the hand or the first report of postoperative pain at the surgical site, whichever occurred earlier. The duration of sensory blockade was defined as the time from the end of the local anesthetic injection to the return of normal pinprick sensation. The duration of the motor block at the elbow and wrist was recorded and was defined as the time from the end of the local anesthetic injection to the return of normal motor strength.

The primary outcome was the duration of analgesia. The secondary outcomes were as follow: (1) the duration of sensory blockade to the musculocutaneous, posterior brachial cutaneous nerve, median nerve, ulnar nerve and radial nerve; (2) the duration of motor block at the elbow and wrist; (3) the mean arterial pressure (MAP) and heart rate (HR) before the anesthesia (T0), at the completion of the anesthesia (T1), at the beginning of the surgery (T2) and at the end of the surgery (T3); and (4) the adverse events recorded, which included excessive sedation, bradycardia, hypotension, respiratory depression, and postoperative anesthesia-related sensory abnormalities in the areas of innervation related to the nerve blocks.

### Sample size calculation

The sample size required for the study was calculated based on the findings of a pilot study performed in our hospital. Five patients were included in each group, and the duration of analgesia was 706 ± 101 min,908 ± 133 min, 732 ± 170 min in the Control group, DexP group, and DexIV group, respectively. Thus, a calculated sample size of 13 patients per group was required to provide a statistical power of 0.80 and a type I error of 0.05 using one-way analysis of variance. The duration of sensory blockade of the musculocutaneous nerve was 160 ± 11 min,200 ± 28 min, 190 ± 35 min in the Control group, DexP group and DexIV group, respectively. Thus, a calculated sample size of 14 patients per group was required to provide a statistical power of 0.80 and a type I error of 0.05 using one-way analysis of variance. The duration of sensory blockade of the posterior brachial cutaneous nerve was 160 ± 11 min,187 ± 42 min, 208 ± 28 min in the Control group, DexP group, and DexIV group, respectively. Thus, a calculated sample size of 16 patients per group respectively was required to provide a statistical power of 0.80 and a type I error of 0.05 using one-way analysis of variance. We expected a prolongation of analgesia and sensory blockade of the musculocutaneous and posterior brachial cutaneous nerve. We assigned 20 patients to each group to allow for possible losses.

### Statistical analysis

SPSS software version 19 (IBM, Armonk, New York) was used for statistical analysis. The Shapiro–Wilk test was used for normality of data distribution. Values for age, height, weight, duration of analgesia, duration of the sensory blockade of the musculocutaneous nerve, posterior brachial cutaneous nerve, median nerve, ulnar nerve and radial nerve, and the duration of motor block of elbow and wrist were presented as mean (SD) and compared using one-way ANOVA, and then the LSD test was used between the two groups when significance was achieved. Values for body mass index (BMI) and surgery time were presented as medians (QR) and were analyzed with the Kruskal–Wallis H test. Values for sex and surgery type were analyzed with the chi-square test or the Fisher exact test. Values for the MAP and HR were compared using repeated-measures ANOVA and Dunnett’s post-tests when significance was achieved. *P* < 0.05 was considered as statistical significance.

## Results

**Fig. 2:**
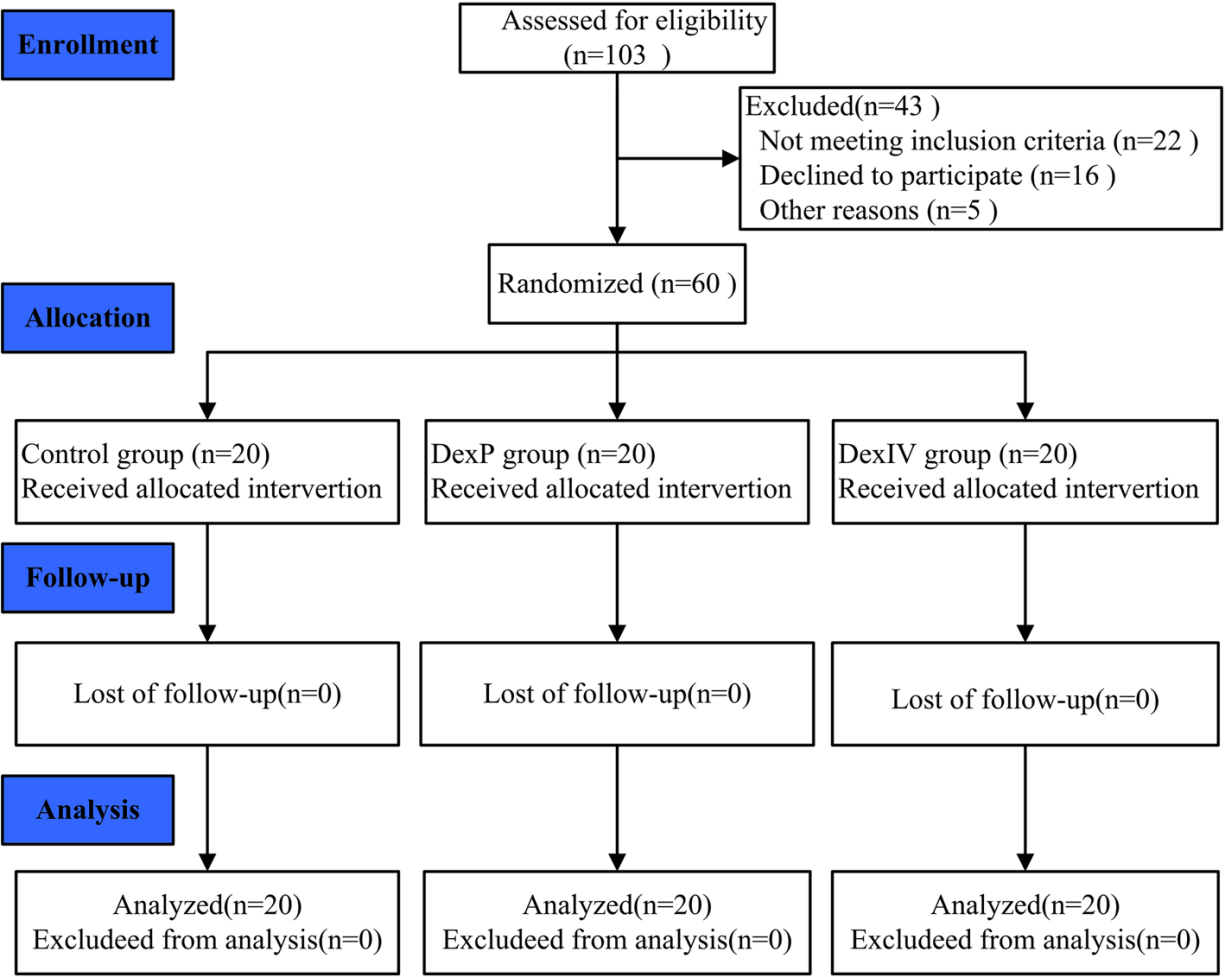
Consolidated Standards of Reporting Trials (CONSORT) flow diagram showing patient progress through the study phases

The CONSORT flow diagram showing patient progress through the study phases is depicted in Fig. 2. The anesthetic effect was satisfactory in all patients, and none of the patient needed conversion to general anesthesia or required additional local anesthetic. No significant differences existed in sex, age, height, weight, BMI, surgery time, or surgery type between the three groups (*P* > 0.05) (Table 1).

**Table 1:** Demographic data and surgery data

Characteristic	Control group	DexP group	DexIV group	*P*
Sex (male/female)	13/7	13/7	12/8	0.871
Age (year)	45 ± 13	47 ± 11	40 ± 14	0.217
Height (cm)	166 ± 7	164 ± 6	166 ± 11	0.685
Weight (kg)	66 ± 10	62 ± 5	63 ± 10	0.260
BMI (kg/m^2)	24(6)	23(1)	22(3)	0.118
Surgery time (min)	42(21)	44(25)	42(28)	0.274
Surgery type				0.402
Tendon anastomosis	4	5	7	
Excision of ganglion cyst	6	4	5	
Metacarpal fracture fixation	10	11	8	

Data are presented as mean ± SD, median (QR) or number, *n* = 20 for all groups

*P* < 0.05 considered statistically significant

The duration of analgesia in the DexP group was significantly longer than that of the other two groups, and there was no difference between the Control and DexIV groups (Table 2).

**Table 2:** Duration of analgesia (min), duration of sensory blockade (min) and duration of motor blockade (min)

End point	Control group	DexP group	DexIV group	*P*	post hoc* analyses*
Duration of analgesia (min)	633 ± 127	997 ± 243	654 ± 159	< 0.001	A, C
Duration of sensory blockade (min)					
musculocutaneous nerve	168 ± 18	197 ± 44	205 ± 57	0.025	A, B
posterior brachial cutaneous nerve	166 ± 17	199 ± 41	196 ± 51	0.015	A, B
median nerve	633 ± 127	997 ± 244	649 ± 164	< 0.001	A, C
ulnar nerve	626 ± 122	996 ± 242	654 ± 159	< 0.001	A, C
radial nerve	633 ± 136	994 ± 243	642 ± 162	< 0.001	A, C
Duration of motor blockade (min)					
elbow	167 ± 17	193 ± 43	194 ± 42	0.034	A, B
wrist	171 ± 17	196 ± 41	196 ± 42	0.047	A, B

Data are presented as mean ± SD, *n* = 20 for all groups. *P* < 0.05 considered statistically significant. A: DexP group differs from Control group (*P* < 0.05). B: DexIV group differs from Control group (*P* < 0.05). C: DexP group differs from DexIV group (*P* < 0.05)

Duration of analgesia: Control group vs. DexP group, *P* < 0.001; Control group vs. DexIV group, *P* = 0.792; DexP group vs. DexIV group, *P* < 0.001

Duration of sensory blockade of musculocutaneous nerve (min): Control group vs. DexP group, *P* = 0.039; Control group vs. DexIV group, *P* = 0.010; DexP group vs. DexIV group, *P* = 0.586

Duration of sensory blockade of posterior brachial cutaneous nerve (min): Control group vs. DexP group, *P* = 0.008; Control group vs. DexIV group, *P* = 0.018; DexP group vs. DexIV group, *P* = 0.755

Duration of sensory blockade of median nerve (min): Control group vs. DexP group, *P* < 0.001; Control group vs. DexIV group, *P* = 0.792; DexP group vs. DexIV group, *P* < 0.001

Duration of sensory blockade of ulnar nerve (min): Control group vs. DexP group, *P* < 0.001; Control group vs. DexIV group, *P* = 0.628; DexP group vs. DexIV group, *P* < 0.001

Duration of sensory blockade of radial nerve (min): Control group vs. DexP group, *P* < 0.001; Control group vs. DexIV group, *P* = 0.874; DexP group vs. DexIV group, *P* < 0.001

Duration of motor block of elbow (min): Control group vs. DexP group, *P* = 0.026; Control group vs. DexIV group, *P* = 0.022; DexP group vs. DexIV group, *P* = 0.941

Duration of motor block of wrist (min): Control group vs. DexP group, *P* = 0.033; Control group vs. DexIV group, *P* = 0.030; DexP group vs. DexIV group, *P* = 0.915

The duration of sensory blockade of the median nerve, ulnar nerve, and radial nerve in the DexP group was significantly longer than that of the other two groups; and there was no difference between the Control and DexIV groups (Table 2).

The duration of sensory blockade of the musculocutaneous and posterior brachial cutaneous nerve in the Control group was significantly shorter than those of the other two groups, but there was no statistically significant difference between the DexIV and DexP groups (Table 2).

The duration of motor blockade at the elbow and wrist joints in the Control group was significantly shorter than that of the other two groups, but there was no statistically significant difference between the DexIV and DexP groups (Table 2).

**Fig. 3:**
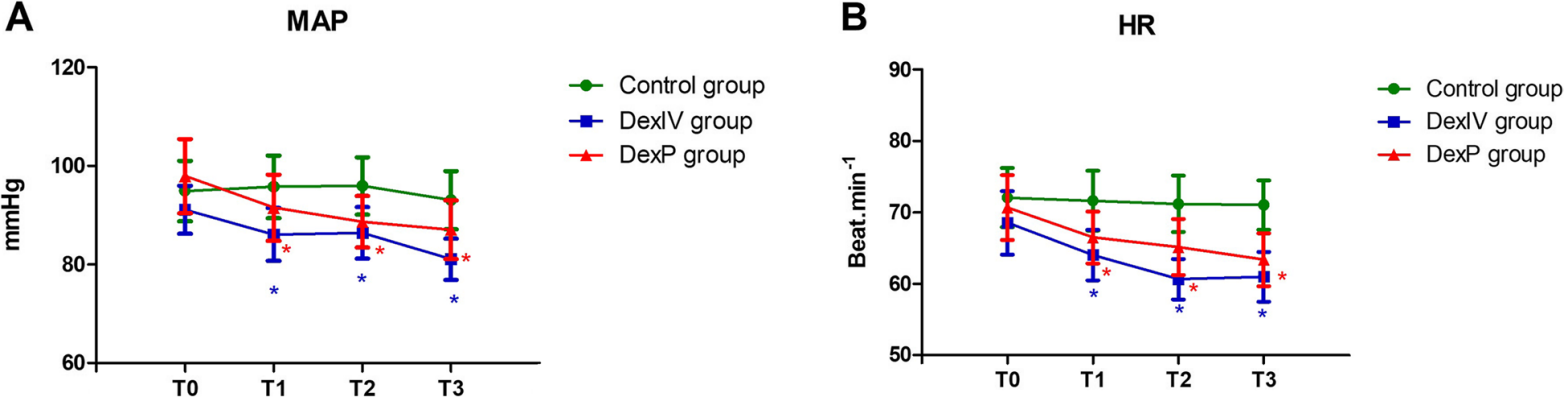
Hemodynamic change during different periods. Values are mean ± IC95%, *n* = 20. * *P* < 0.05, versus T0 value within the group

Figure 3 illustrates the hemodynamic changes during different periods. Significant differences were seen in MAP and HR during different periods among the three groups. The MAP and HR were significantly decreased in the DexIV and DexP groups at different points in time. The MAP and HR at the completion of anesthesia (T1), at the beginning of the surgery (T2), and at the end of the surgery (T3) were decreased compared with the basic value (T0) in the DexIV and DexP groups. There was no significant decrease in the Control group at any point in time.

No patient in any of the groups experienced any adverse events, including excessive sedation, bradycardia, hypotension, respiratory depression, and postoperative anesthesia-related sensory abnormalities in the areas of innervation related to the nerve blocks.

## Discussion

In this trial, peripheral dexmedetomidine was observed to prolong the duration of analgesia for hand surgery after a mid-forearm nerves block with 0.75% ropivacaine, whereas IV dexmedetomidine exerted no such effect. IV and perineural dexmedetomidine similarly prolonged the duration of motor blockade of the upper limb. Additionally, adding dexmedetomidine to a 0.75% ropivacaine mid-forearm nerves block prolonged the duration of a 1.5% lidocaine supraclavicular brachial plexus block, and IV dexmedetomidine prolonged the supraclavicular lidocaine block; the effects were comparable. Hence, there should be a primary peripheral mechanism and a secondary systemic mechanism when dexmedetomidine is used as an adjuvant to local anesthetics.

The addition of dexmedetomidine to a local anesthetic for a brachial plexus block has been shown to prolong the duration of the block and that of postoperative analgesia [9, 10]. Studies have suggested that both perineural and IV administration are effective [1, 11]. However, whether the effectiveness of perineural dexmedetomidine is exerted via a perineural effect or a systemic effect was unclear. Systemic mechanisms [12–14] have been proposed to explain the prolongation of the effect when using dexmedetomidine used as an adjuvant to local anesthetics, which may suggest that different routes of administration may produce similar effects. Abdallah et al [15] compared the efficacy of IV with the perineural administration of 0.5 µg/kg of dexmedetomidine in prolonging the duration of analgesia for outpatient shoulder surgery after interscalene brachial plexus block with 15 mL of 0.5% ropivacaine. The investigators reported the duration of analgesia to be 10.9 h with perineural dexmedetomidine and 9.8 h with IV dexmedetomidine; both were longer than local anesthetic alone (6.7 h). They demonstrated that IV and perineural dexmedetomidine exhibited similar effects in prolonging the duration of analgesia for outpatient shoulder surgery after interscalene brachial plexus block. The observed prolongation in the two dexmedetomidine groups could be attributed to the absorption and redistribution of the perineural dexmedetomidine, which triggered systemic effects.

A systematic review [1] examining 10 trials comparing IV and perineural dexmedetomidine for peripheral nerve block concluded that IV dexmedetomidine appears to be an inferior peripheral nerve block adjunct compared with perineural dexmedetomidine. A volunteer study by Marhofer et al [16] showed that peripheral or IV use of dexmedetomidine can prolong the duration of an ulnar nerve block; while perineural dexmedetomidine prolonged the block duration by 60%, IV administration prolonged it by 10%. Another study [17] reported a prolongation of 30% and 100% of ulnar nerve blocks with IV and perineural treatments, respectively, compared with an identical dose of ropivacaine alone. These findings indicate that the longer duration of nerve block with perineural dexmedetomidine may be caused by a perineural mechanism. A systematic review [5] suggested that IV dexmedetomidine does not prolong sensory, motor, or analgesic block durations, which is consistent with our finding that IV dexmedetomidine does not affect the sensory block or analgesic duration. In our results, the duration of analgesia and the sensory block durations of the median, ulnar, and radial nerves (related to the effect of the mid-forearm nerve blocks) in the DexP group were significantly longer than that of Control group and DexIV group, and there was no difference between the two groups. We demonstrated that adding dexmedetomidine to ropivacaine mid-forearm nerve blocks could prolong the duration of a mid-forearm nerve block, whereas the IV use of dexmedetomidine did not prolong the duration of mid-forearm nerve blocks. Hence, dexmedetomidine does appear to have a peripheral mechanism.

As a selective α2 adrenoceptor agonist, dexmedetomidine produces centrally mediated analgesic effects in the brain and spinal cord via the alpha 2 receptor [18], which explains the systemic mechanism of the analgesic effect of dexmedetomidine. Some evidence has indicated [19] that dexmedetomidine plays an inhibitory role in delayed rectifier K + current and Na + current, which reduces neuronal activity. In animal experiments, dexmedetomidine has been reported to prolong the peripheral nerve block by blocking the hyperpolarization-activated cation current, thereby delaying the restoration of resting potential and preventing the conduction of a new action potential, which is considered to be the peripheral mechanism of dexmedetomidine [20]. This effect seems to be more pronounced in C fibers (pain) than in A fibers (motor). Therefore, the analgesic effect of dexmedetomidine may be more pronounced than the motor response. Hence, in our trial, the difference in the duration of analgesia was more notable than that of the motor block between the DexP and DexIV groups.

The model in our study involved a lidocaine supraclavicular brachial plexus block combined with ropivacaine mid-forearm nerve blocks. Dexmedetomidine was either added to the ropivacaine or was given IV, and compared with the Control group. The results showed that the duration of sensory blockade of the musculocutaneous and posterior brachial cutaneous nerve (indicating the effect of the supraclavicular brachial plexus block) in the DexP and DexIV groups were both significantly longer than those of the Control group; there was no difference between DexP group and DexIV group. This finding established that adding dexmedetomidine to ropivacaine mid-forearm nerve blocks can not only prolong the duration of mid-forearm nerve blocks but also prolong the duration of supraclavicular brachial plexus block. Furthermore, the prolonged effect of the lidocaine supraclavicular brachial plexus block was similar with IV use of dexmedetomidine. Therefore, a systemic mechanism may also be involved when dexmedetomidine is used as a local anesthetic adjuvant for lidocaine supraclavicular brachial plexus block.

Dexmedetomidine has not been approved for use by the US Food and Drug Administration as a local anesthetic adjuvant. The issue of clinical safety, therefore, needs to be examined. In this study, we did not observe serious adverse events, such as severe bradycardia, severe hypotension and nerve injury. The preclinical data [21, 22] suggest that peripheral dexmedetomidine may offer neuroprotective effects via the anti-inflammatory effect of alpha-2 adrenoceptor agonists that attenuate the inflammatory response when local anesthetics are applied perineurally. However, more clinical data are needed. It is worth noting that in our results MAP and HR decreased to some extent after IV and peripheral administration of dexmedetomidine. We speculate that the systemic mechanism of dexmedetomidine is related to hemodynamic changes. The decrease in blood pressure and HR may lower the blood flow to the heart, muscles, and liver, which may, in turn, slow down the elimination and metabolism of peripheral ropivacaine and lidocaine. Decreased drug clearance because of a reduction of HR and MAP has often been demonstrated during general anesthesia [23]. This effect is more pronounced in drugs with a short half-life than those with a long half-life. This difference may explain why peripheral dexmedetomidine was ineffective on distal nerve blocks with a long-acting local anesthetic (ropivacaine), but significantly prolonged the supraclavicular brachial plexus block with a short-acting local anesthetic (lidocaine). However, this assumption is speculative because plasma levels of dexmedetomidine, ropivacaine or lidocaine were not measured. The systemic mechanism of how dexmedetomidine works to prolong the duration of blockade when added to a local anesthetic remains to be confirmed via further research.

This study has certain limitations. The concentration of dexmedetomidine in the blood was not measured. Owing to the absence of a plasma dexmedetomidine measurement after its administration with ropivacaine in the forearm, the proportion of ropivacaine systemic reabsorption could not be quantified. Moreover, the relationship between the plasma concentration and the prolonged duration of the blockade after the absorption of peripheral dexmedetomidine was not determined. In future studies, we intend to delineate the effect of dexmedetomidine on the pharmacokinetics of local anesthetics.

## Conclusions

In conclusion, perineural dexmedetomidine prolongs the analgesic effect of ropivacaine in mid-forearm nerve blocks through a primary peripheral mechanism. A secondary systemic mechanism of dexmedetomidine in prolonging the blockade duration of the lidocaine supraclavicular block also exists.

## Data Availability

Data are available upon reasonable request. The technical appendix, statistical code and data set are available from the corresponding author.

## Abbreviations

IV: Intravenous
ASA: American Society of Anesthesiologists
NRPS: Numerical Rating Pain score
MAP: Mean arterial pressure
HR: Heart rate
BMI: Body mass index

## References

[1] Hussain N, Brummett CM, Brull R (2021). Efficacy of perineural versus intravenous dexmedetomidine as a peripheral nerve block adjunct: a systematic review. Reg Anesth Pain Med.

[2] Bansal P, Garg S (2019). Effect of Adding Dexmedetomidine to Local Anesthetic Agents for Transversus Abdominis Plane Block: A Meta-analysis. Clin J Pain.

[3] Sun Q, Liu S, Wu H (2019). Dexmedetomidine as an Adjuvant to Local Anesthetics in Transversus Abdominis Plane Block: A Systematic Review and Meta-analysis. Clin J Pain.

[4] Vorobeichik L, Brull R, Abdallah FW (2017). Evidence basis for using perineural dexmedetomidine to enhance the quality of brachial plexus nerve blocks: a systematic review and meta-analysis of randomized controlled trials. Br J Anaesth.

[5] Sehmbi H, Brull R, Ceballos KR (2021). Perineural and intravenous dexamethasone and dexmedetomidine: network meta-analysis of adjunctive effects on supraclavicular brachial plexus block. Anaesthesia.

[6] Somsunder R, Archana N, Shivkumar G (2019). Comparing efficacy of perineural dexmedetomidine with intravenous dexmedetomidine as adjuvant to levobupivacaine in supraclavicular brachial plexus block. Anesth Essays Res.

[7] Nana B, Limei C, Yun X (2018). Effect of ultrasound-guided nerve block with 0.75% ropivacaine at the mid-forearm on the prevalence of moderate to severe pain after hand surgery. Clin Ther.

[8] Tran DQ, Muñoz L, Zaouter C (2009). A prospective, randomized comparison between single-and double-injection, ultrasound-guided supraclavicular brachial plexus block. Reg Anesth Pain Med.

[9] Koraki E, Stachtari C, Kapsokalyvas I, Trikoupi A (2018). Dexmedetomidine as an adjuvant to 0.5% ropivacaine in ultrasound-guided axillary brachial plexus block. J Clin Pharm Ther.

[10] Nallam SR, Chiruvella S, Karanam S (2017). Supraclavicular brachial plexus block: Comparison of varying doses of dexmedetomidine combined with levobupivacaine: A double-blind randomised trial. Indian J Anaesth.

[11] Motaghi E, GhasemiPirbalooti M, Bozorgi H (2021). Safety and Efficacy of Dexmedetomidine in Breast Surgeries: A Systematic Review and Meta-Analysis. J Perianesth Nurs.

[12] Jorm CM, Stamford JA (1993). Actions of the hypnotic anaesthetic, dexmedetomidine, on noradrenaline release and cell firing in rat locus coeruleus slices. Br J Anaesth.

[13] Guo TZ, Jiang JY, Buttermann AE, Maze M (1996). Dexmedetomidine injection into the locus ceruleus produces antinociception. Anesthesiology.

[14] Weinbroum AA, Ben-Abraham R (2001). Dextromethorphan and dexmedetomidine: new agents for the control of perioperative pain. Eur J Surg.

[15] Abdallah FW, Dwyer T, Chan VW (2016). IV and Perineural Dexmedetomidine Similarly Prolong the Duration of Analgesia after Interscalene Brachial Plexus Block A Randomized, Three-arm, Triple-masked. Placebo-controlled Trial Anesthesiology.

[16] Marhofer D, Kettner SC, Marhofer P (2013). Dexmedetomidine as an adjuvant to ropivacaine prolongs peripheral nerve block: a volunteer study. Br J Anaesth.

[17] Andersen JH, Jaeger P, Grevstad U (2019). Systemic dexmedetomidine is not as efficient as perineural dexmedetomidine in prolonging an ulnar nerve block. Reg Anesth Pain Med.

[18] Carollo DS, Nossaman BD, Ramadhyani U (2008). Dexmedetomidine: a review of clinical applications. Curr Opin Anaesthesiol.

[19] Chen BS, Peng H, Wu SN (2009). Dexmedetomidine, an alpha2- adrenergic agonist, inhibits neuronal delayed-rectifier potassium current and sodium current. Br J Anaesth.

[20] Brummett CM, Hong EK, Janda AM (2011). Perineural dexmedetomidine added to ropivacaine for sciatic nerve block in rats prolongs the duration of analgesia by blocking the hyperpolarization-activated cation current. Anesthesiology.

[21] Brummett CM, Norat MA, Palmisano JM (2008). Perineural administration of dexmedetomidine in combination with bupivacaine enhances sensory and motor blockade in sciatic nerve block without inducing neurotoxicity in rat. Anesthesiology.

[22] Brummett CM, Padda AK, Amodeo FS (2009). Perineural dexmedetomidine added to ropivacaine causes a dose-dependent increase in the duration of thermal antinociception in sciatic nerve block in rat. Anesthesiology.

[23] Mertens MJ, Olofsen E, Burm AG (2004). Mixed-effects modeling of the influence of alfentanil on propofol pharmacokinetics. Anesthesiology.

